# Effects of Aflatoxin B_1_ on T-Cell Subsets and mRNA Expression of Cytokines in the Intestine of Broilers

**DOI:** 10.3390/ijms16046945

**Published:** 2015-03-27

**Authors:** Min Jiang, Xi Peng, Jing Fang, Hengmin Cui, Zhengqiang Yu, Zhengli Chen

**Affiliations:** 1 Key Laboratory of Animal Diseases and Environmental Hazards of Sichuan Province, Ya’an 625014, Sichuan, China; E-Mails: minjiangchn@hotmail.com (M.J.); pengxi197313@163.com (X.P.); cuihengmin@sina.com (H.C.); mine_yzq@163.com (Z.Y.); 2 College of Veterinary Medicine, Sichuan Agricultural University, Ya’an 625014, Sichuan, China; E-Mail: chzhli75@163.com

**Keywords:** aflatoxin B_1_, T-cell subsets, cytokine, intestinal mucosal immunity, broilers

## Abstract

This study was conducted to investigate the effects of aflatoxin B_1_ (AFB_1_) on T-cell subsets and mRNA expression of cytokines in the small intestine of broilers. One hundred and fifty-six one-day-old healthy Cobb broilers were randomly divided into control group (0 mg/kg AFB_1_) and AFB_1_ group (0.6 mg/kg AFB_1_) with three replicates per group and 26 birds per replicate for 21 days, respectively. At 7, 14, and 21 days of age, the duodenum, jejunum and ileum were sampled for analyzing T cell subsets (CD3^+^, CD3^+^CD4^+^ and CD3^+^CD8^+^) by flow cytometry as well as IL-2, IL-4, IL-6, IL-10, IL-17, IFN-γ and TNF-α mRNA expression by qRT-PCR. The percentages of T-cells in the intra-epithelial lymphocytes (IELs) and lamina propria lymphocytes (LPLs) of duodenum, jejunum and ileum in the AFB_1_ group showed a decreased tendency in comparison to the control group. The mRNA expression of cytokines in the three intestinal segments in the AFB_1_ group presented a general decline compared with the control groups. Our data demonstrated that 0.6 mg/kg AFB_1_ in the broilers diet could reduce the percentages of T-cell subsets and the expression level of cytokine mRNA in the small intestine, implying that the immune function of the intestinal mucosa might be affected. The reduction of cytokines mRNA expression may be closely associated with the decreased proportions of T cells subsets induced by AFB_1_.

## 1. Introduction

Aflatoxins are mainly produced by *Aspergillus flavus* and *A. parasiticus* and are found in various agricultural commodities [1]. Of the known aflatoxins, aflatoxin B_1_ (AFB_1_) is the most toxic congener, and has potent hepatotoxicity, carcinogenicity, cytotoxicity, genotoxicity and immunotoxicity [2,3]. Aflatoxins are deleterious to poultry and their contamination in feed is practically unavoidable. The United States Food and Drug Administration regulates the amount of permissable AFB_1_ in poultry feed. Current action levels for corn and peanut products is 0.1 mg/kg, and for cottonseed meal is 0.3 mg/kg [4].

Previous research studies have indicated that AFB_1_ has immunosuppressive action and affects humoral and cellular responses [5,6], especially the latter [7]. Exposure of animals to AFB_1_ (0.005–0.075 mg/kg body weight) for one week showed dose-dependent decreases in the percentage of splenic CD8^+^ T cells and CD3^−^CD8a^+^ natural killer (NK) cells [7]. Mice orally treated with 0.2 mg/kg AFB_1_ showed declined numbers of CD3^+^ in intestine [8]. In chick, 0.3 mg/kg dietary AFB_1_ has also been shown to decrease the percentages of CD3^+^, CD3^+^CD4^+^ and CD3^+^CD8^+^ T cells in the peripheral blood, thymus and spleen [9,10,11].

Although many data on the effect of AFB_1_ on immune cell functions are available, few studies have investigated their effects on cytokine expression, and the results are somewhat conflicting. An impact of AFB_1_ on the production of interleukin-2 (IL-2) by splenocytes and interleukin-1 (IL-1) by peritoneal macrophages was reported in rats injected with 1 mg/kg AFB_1_ body weight [12]. Exposure of rats to AFB_1_ (0.005–0.075 mg/kg body weight), a general influence on the expression of interleukin-4 (IL-4), interferon γ (IFN-γ) and tumor necrosis factor α (TNF-α) in rat splenocytes was also found [13]. Suppressive effects of inflammatory cytokines were observed in rats/mice during respiratory aflatoxicosis [14]. In chick, 0.3 mg/kg dietary AFB_1_ could reduce the contents of serum IL-2 and IFN-γ [10]. However, increased levels of interleukin-6 (IL-6), IFN-γ and TNF-α proteins and mRNA expression in serum and spleen were also reported in broilers fed with diets containing 0.074 mg/kg AFB_1_ [15].

Following ingestion of AFB_1_-contaminated food or feed, the intestine could be exposed to a high concentration of toxin. This organ is not only a physical barrier but also an active component of the mucosal immune system, playing an important role in cellar immunity [16]. Various T-lymphocyte subpopulations in the intestine are accumulated in intra-epithelium (IEL) and the lamina propria (LP) [17]. Many functions of T cells in the gastrointestinal immune system are mediated by secreted cytokines [18]. In contrast to systemic AFB_1_ immunotoxicity, limited information is available concerning the influence of AFB_1_ on the intestinal mucosal cytokine mRNA expression and T cell subsets induced by dietary AFB_1_.

The aims of the present study were to investigate the effects of 0.6 mg/kg dietary AFB_1_ on the expression of IL-2, IL-4, IL-6, interleukin-10 (IL-10), interleukin-17 (IL-17), IFN-γ and TNF-α mRNA by quantitative real-time PCR (qRT-PCR) and proportions of T-cell subsets by flow cytometry (FCM) in broilers’ small intestine. The results could provide new experimental evidence for evaluating intestinal mucosal immunity and understanding the mechanisms of the immunosuppressive effects induced by dietary AFB_1_ in broilers.

## 2. Results

### 2.1. T-Cell Subsets in the Small Intestinal Intraepithelial Lymphocytes (IELs)

**Figure 1:**
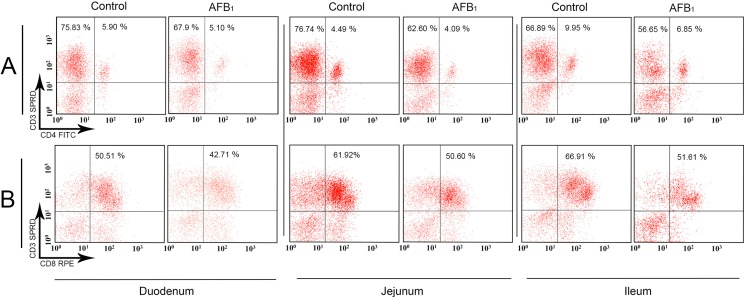
The quadrantal diagram of the intraepithelial (duodenal, jejunal and ileum) CD3^+^, CD3^+^CD4^+^ and CD3^+^CD8^+^ IELs T-cell percentages in the control and AFB_1_ groups at 21 days of age. The numbers in each quadrant indicate the percentage of cells. **Panel A**: CD3^+^CD4^+^ IELs T-cell in the upper right, and CD3^+^ IELs T-cell in the upper right and upper left; **Panel B**: CD3^+^CD8^+^ IELs T-cell in the upper right.

**Figure 2:**
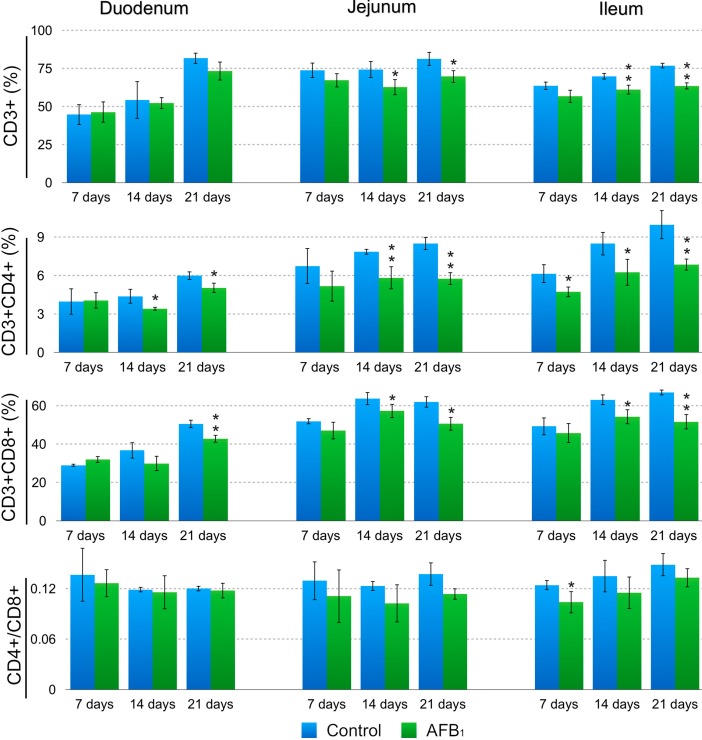
Changes of the small intestinal CD3^+^, CD3^+^CD4^+^, and CD3^+^CD8^+^ IELs T-cell percentages and CD4^+^/CD8^+^ ratios at 7, 14 and 21 days of age. Note: Data are presented with the means ± standard deviation (*n* = 6). * *p* < 0.05, ** *p* < 0.01.

Significant decreases in the CD3^+^ IELs percentages in the AFB_1_ group were observed in the jejunum (*p* < 0.05) and ileum (*p* < 0.01) at 14 and 21 days of age and not in the duodenum (*p* > 0.05) in comparison to the control group. The percentages of the CD3^+^CD4^+^ IELs in the AFB_1_ group were significantly decreased in the duodenum (*p* < 0.05) and jejunum (*p* < 0.01) at 14 and 21 days, and in the ileum at 7, 14 and 21 days (*p* < 0.05 or 0.01). The percentages of the CD3^+^CD8^+^ IELs in the AFB_1_ group were dramatically dropped in the duodenum (*p* < 0.01) at 21 days, in the jejunum and ileum at 14 (*p* < 0.01) and 21 days (*p* < 0.05 or 0.01). A significant decrease in the IELs CD4^+^/CD8^+^ ratio in the AFB_1_ group was observed in the ileum at 7 days (*p* < 0.05) and not in the duodenum and jejunum (*p* > 0.05); detailed results are shown in Figure 1 and Figure 2.

### 2.2. T-Cell Subsets in the Small Intestinal Lamina Propria Lymphocytes (LPLs)

**Figure 3:**
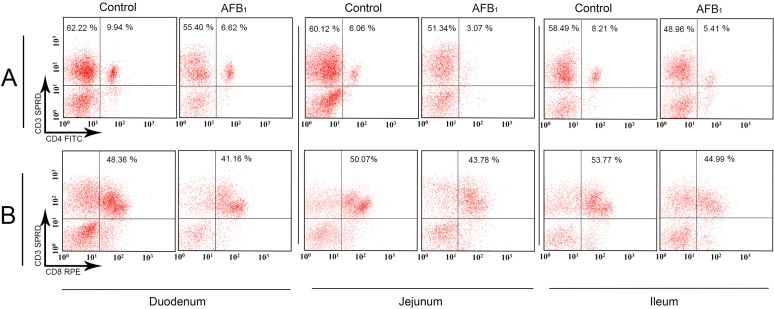
The quadrantal diagram of the lamina propria (duodenal, jejunal and ileum) CD3^+^, CD3^+^CD4^+^ and CD3^+^CD8^+^ LPLs T-cell percentages in the control and AFB_1_ groups at 21 days of age. The numbers in each quadrant indicate the percentage of cells. **Panel A**: CD3^+^CD4^+^ LPLs T-cell in the upper right, and CD3^+^ LPLs T-cell in the upper right and upper left; **Panel B**: CD3^+^CD8^+^ LPLs T-cell in the upper right.

**Figure 4:**
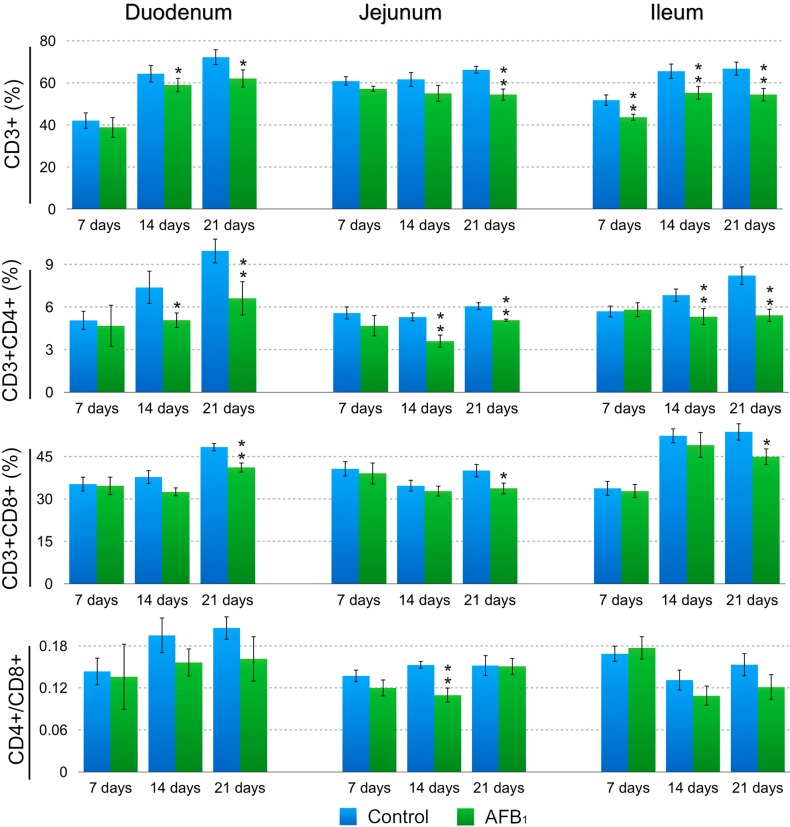
Changes of the small intestinal CD3^+^, CD3^+^CD4^+^, and CD3^+^CD8^+^ LPLs T-cell percentages and CD4^+^/CD8^+^ ratios at 7, 14 and 21 days of age. Note: Data are presented with the means ± standard deviation (*n* = 6). * *p* < 0.05, ** *p* < 0.01.

When compared with those of the control group, the percentages of the CD3^+^ LPLs in the AFB_1_ group were significantly decreased in the duodenum at 14 and 21 days of age (*p* < 0.05), in the jejunum at 21 days (*p* < 0.01) and in the ileum at 7, 14 and 21 days (*p* < 0.01). The percentages of the CD3^+^CD4^+^ LPLs in the AFB_1_ group dramatically dropped in three intestinal segments at 14 and 21 days of age (*p* < 0.05 or 0.01). The significant declined percentages of the CD3^+^CD8^+^ LPLs in the AFB_1_ group were seen in the three intestinal segments only at 21 days of age (*p* < 0.01 or 0.05). A significant decrease in the LPLs CD4^+^/CD8^+^ ratio in the AFB_1_ group was observed in the jejunum only at 14 days (*p* < 0.01) and not in the duodenum and ileum (*p* > 0.05); detailed results are shown in Figure 3 and Figure 4.

### 2.3. The Expression Levels of the IL-2, IL-4, IL-6, IL-10, IL-17, IFN-γ and TNF-α (LITAF) mRNA in the Small Intestinal Mucosa

**Figure 5:**
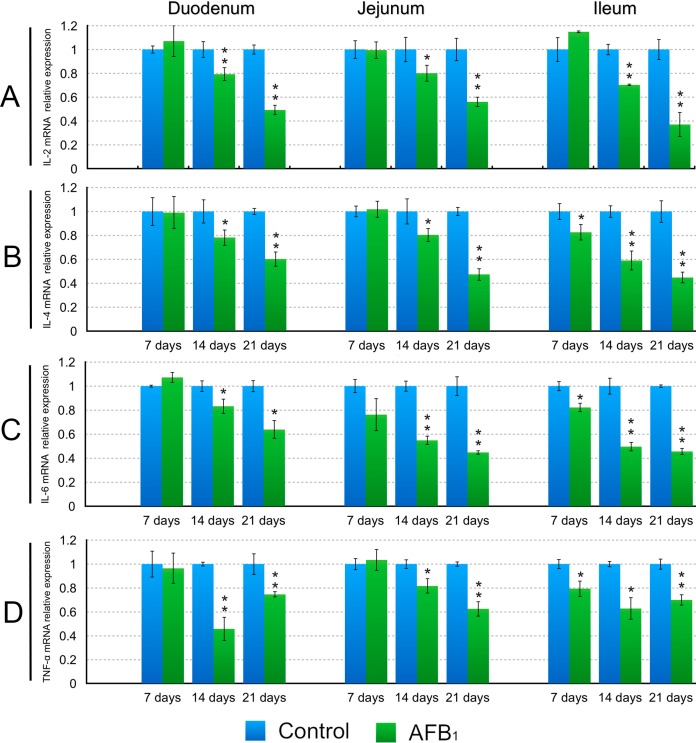
Levels of the IL-2 (**A**); IL-4 (**B**); IL-6 (**C**) and TNF-α (LITAF) (**D**) mRNA expression in the small intestine. Data are presented with the means ± standard deviation (*n* = 6). * *p* < 0.05, ** *p* < 0.01.

**Figure 6:**
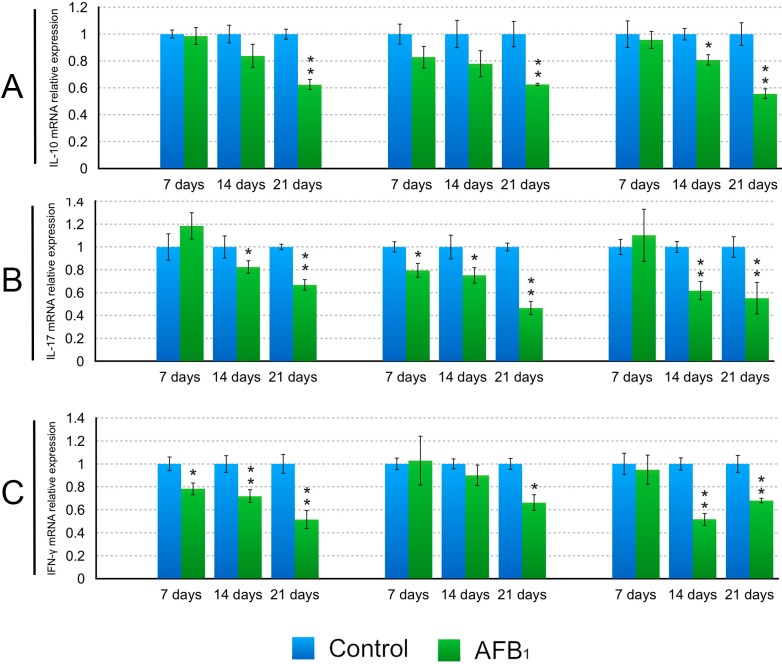
Levels of the IL-10 (**A**); IL-17 (**B**) and IFN-γ (**C**) mRNA expression in the small intestine. Data are presented with the means ± standard deviation (*n* = 6). * *p* < 0.05, ** *p* < 0.01.

The mRNA expression levels of the IL-2, IL-4, IL-6 and TNF-α mRNA in the AFB_1_ group showed significant decreases in the duodenum and jejunum at 14 and 21 days of age (*p* < 0.05 or 0.01), and in the ileum at 7, 14 and 21 days (*p* < 0.05 or 0.01) in comparison to the control group except for the IL-2 at 7 days of age (*p* > 0.05) (Figure 5). The significant decreases of the IL-10 mRNA expression levels in the AFB_1_ group were observed in the duodenum and jejunum at 21 days of age (*p* < 0.01), and in the ileum at 14 and 21 days (*p* < 0.05 or 0.01) (Figure 6). The IL-17 mRNA expression levels in the AFB_1_ group presented dramatic decline in the three intestinal segments (*p* < 0.05 or 0.01) during the experiment except for the duodenum and ileum at 7 days of age (*p* > 0.05) (Figure 6). The significant decreases of the IFN-γ mRNA expression in the AFB_1_ group were seen in the duodenum at 7, 14 and 21 days of age, and in the jejunum at 21 days, and in the ileum at 14 and 21 days of age (*p* < 0.05 or 0.01) (Figure 6).

## 3. Discussion

Resident T cells represent a major component of the gut mucosa and play an important role in the mucosa cellular immunity [19]. The CD3^+^ molecules are the surface marker of mature T cells. The CD4^+^ T-cells are considered as helper/inflammatory T-cells which respond to exogenous antigens in conjunction with the class II major histocompatibility complex (MHC-II) molecules, and CD8^+^ T-cells respond to endogenous antigen in association with MHC-I molecules and generally function as cytotoxic T-cells. As shown in our results, the percentages of T-cell subsets (CD3^+^, CD3^+^CD4^+^, and CD3^+^CD8^+^) in the IELs and LPLs of duodenum, jejunum and ileum presented a decreased tendency in AFB_1_ group compared to the control group. These results indicated that 0.6 mg/kg dietary AFB_1_ could reduce the population of T-cell subsets, and then impact the cellular immunity of broilers’ intestine. Our observations are similar to Tomkova’s report in which the mucosa of the intestine showed a significant decline in the number of CD3^+^ T cells in 0.2 mg/kg AFB_1_ treated mice [8]. Developed and differentiated in bone marrow and thymus, T cells migrate into the blood circulation, and then the intestinal mucosa. Early studies have shown that AFB_1_ can induce: the suppression of myelopoesis in the bone marrow [8]; The decrease in thymic relative weight; The increase in apoptotic thymocytes [9]; The decline in lymphocytes in the peripheral blood [10]; And the damage of enterocytes [8,20]. Thus, the decreased proportion of intestinal T-cell subsets in AFB_1_-treated broilers observed in our experiment can be explained by disturbances in bone marrow function and impairment in thymus development, and these may lead to a systemic decrease in immunocompetent cells in the blood circulation and subsequently in the intestine. Another possibility was distribution of the signal transduction transfer from functional damaged enterocytes to IELs and LPLs [8].

Huang *et al.* reported that in chicken there were only a few CD4^+^ T cells in the intestine while CD8^+^ T cells predominate [21]. Similar to Huang’s report, we also found that CD4^+^ T cells represented minor proportions among the intestinal lymphocyte subpopulations since CD4^+^/CD8^+^ ratios both in IELs and LPLs were less than 1.0 in all samples of both groups. In this research, CD4^+^/CD8^+^ ratios in LPLs and LPLs of AFB_1_ group showed a decreased tendency during the experiment compared with control group. However, only jejunal CD4^+^/CD8^+^ ratio in LPLs at 14 days of age and the ileum CD4^+^/CD8^+^ ratio in IELs at 7 days in the AFB_1_ group were significantly lower, suggesting that 0.6 mg/kg AFB_1_ may affect CD4^+^ T cells more effectively than CD8^+^ T cells because the CD8^+^ T cell population was not significantly decreased (see Figure 2 and Figure 4). The ratios in the duodenum at 7, 14 and 21 days and in the jejunum at 7 and 21 days as well as in the ileum at 14 and 21 days were not significantly decreased, indicating that the relative proportions of these T-cell subpopulations were maintained because the percentages of CD3^+^CD4^+^ and CD3^+^CD8^+^ molecules declined at the same time. Our above results also indicated that the effects on the CD4^+^/CD8^+^ ratios caused by AFB_1_ varied for different intestinal segments and different times. The reason for this variation is unknown and needs further study. However, it is interesting to note that in chick, the composition of various T cells subsets in the intestine depends upon host age, the regions of gut examined and the genetic background of the host [17].

It has been noted that AFB_1_ performs part of its immunosuppressive effects through some cytokines [22]. Meanwhile, early research revealed that AFB_1_ can lead to the alteration of cytokine expression and production [6,7,13,14,15,23,24]. In the present study, a general decline of the IL-2, IL-4, IL-6, IL-10, IL17, IFN-γ and TNF-α mRNA expression in the duodenum, jejunum and ileum was found in the AFB_1_ group. Similarly, it was reported that exposure of rats to AFB_1_ (0.005–0.075 mg/kg body weight), resulted in a decreased expression of IL-4, IFN-γ and TNF-α in rat splenocytes [13]. Other researchers demonstrated that AFB_1_ inhibited the mRNA and protein expression of IL-4, IL-6 and IL-10 from peritoneal macrophages, splenic lymphocytes and macrophage cell lines [6,23,24], and suppressed the effects of the IFN-γ and TNF-α in rats and mice during respiratory aflatoxicosis [14]. Contrary to the above results, a significant up-regulation of the IL-1, IL-6, IL-10, IFN-γ and TNF-α mRNA expression was observed in spleen from pigs exposed to dietary 1.807 mg/kg of AFB_1_ [7]. In addition, 0.074 mg/kg dietary AFB_1_ could induce an increase in the IL6, TNF-α and IFN-γ proteins and mRNA expression in the broilers’ serum and spleen [15]. Thus, the effects of AFB_1_ on cytokines are not conclusive, which might be attributed to the type, the dose, the duration of exposure, the susceptibility of each tissue and animal species as well as other experimental conditions.

Cytokines are important determinants and modulators of immune function and play a critical role in innate and adopted immunity of gut mucosa. IL-2, also referred to as T-cell growth factor, produced by activated T lymphocytes, modulates the B and T-lymphocyte functions [25]. IFN-γ, TNF-α and IL-6 are typical examples of multifunctional cytokines involved in the regulation of the immune response, hematopoiesis, and inflammation. Their functions are widely overlapping but each shows its own characteristic properties. IFN-γ, mainly produced by T cells and NK cells, is a regulator of numerous immunological functions such as immunomodulation and leukocyte trafficking [26]. During the inflammation, IFN-γ acts to modify epithelial and endothelial barrier function [27]. TNF-α, which is secreted by activated T cells, has the ability to increase intestinal permeability [28]. IL-4 plays a key role in the host’s protective immunity [29]. IL-6 was originally identified as a B-cell differentiation factor, and one of its major functions is antibody induction [30]. IL-10 is a pleiotropic cytokine produced mainly by activated T cells, B cells, monocytes/macrophages [31], and plays an important immunoregulatory role in the intestine, especially as a differentiation factor for a novel subset of T cells with suppressor function [31]. IL-17 is produced mainly by activated T cells, and it induces the production of IL-6 as well as takes part in orchestrating the gut mucosal barrier against gastrointestinal pathogens [19]. Based on all this information, the decreased mRNA expression of cytokines in this study suggested that 0.6 mg/kg dietary AFB_1_ might impair the immune function of the intestinal mucosa in broilers chicken. One of the mechanisms for this may be attributed to the declined proportions of the T cell subsets induced by dietary AFB_1_ observed in the present experiment because these cytokines were mainly produced by T cells, and one of the main ways that cytokines exert key effects on immune responses is determined by the differentiation of T-cell subsets [32].

Mycotxins including aflatoxins are known to affect intestinal function and health, and development [33]. Yunus reported that during week four of exposure under 0.75 mg of AFB_1_/kg of diet, a linear decrease in unit weight appeared to progress from the proximal (duodenum) to the distal (jejunum) small intestine with increase in the length of exposure in broilers, while a compensatory linear increase in the length of the duodenum and jejunum was noted [34]. This study indicated that effects of AFB_1_ on intestinal morphology could differ with progression of the exposure. In our research reported here, it is interesting to note that during week three of exposure, AFB_1_ significantly altered T-cell subset proportions and mRNA expression of cytokines in the duodenum, jejunum and ileum mostly at 14 and 21 days of age, suggesting that such effect could differ with progression of the exposure. Further studies, however, are needed to unveil the underlying mechanisms.

## 4. Experimental Section

### 4.1. Animals and Diets

One hundred and fifty-six one-day-old healthy Cobb broilers purchased from the Chia Tai Group (Wenjiang, Sichuan, China), were randomly divided into control group (0 mg/kg AFB_1_) and AFB_1_ group (0.6 mg/kg AFB_1_) with three replicates per group and 26 birds per replicate. The basal diet, namely the control diet, was formulated according to National Research Council (NRC, 1994) [35] and Chinese Feeding Standard of Chicken (NY/T33-2004) recommendations. The AFB_1_-contaminated diet was made, similarly to the method described by Kaoud [36]. Briefly, 27 mg AFB_1_ (Sigma-Aldrich, Saint Louis, MO, USA) was dissolved into 30 mL methanol, and then the 30-mL mixture was mixed into the 45-kg corn-soybean basal diet to formulate the AFB_1_-contaminated diet. The equivalent methanol was mixed into corn-soybean basal diet to produce the control diet. Then the methanol in the diets was evaporated at 98 °F (37 °C). After preparing the diet, the control and AFB_1_-contaminated diets were analyzed by HPLC (Waters, Milford, MA, USA) and fluorescence detector (Waters, Model 2475, Milford, MA, USA) method to ensure the AFB_1_ concentration in the experimental diets. Aflatoxin B_1_ content was 0.601 mg/kg in the contaminated diet and less than 0.001 mg/kg in the control diet. Broilers were housed in cages with electrically heated units and provided with water as well as diet (specified in Table 1) *ad libitum* for 21 days. The animal protocols used in this work and all procedures of the experiment were performed in compliance with the laws and guidelines of Sichuan Agricultural University Animal Care and Use Committee (Approval No: 2012-024).

**Table 1:** Composition of the basal diet.

Composition	Content (%)	Nutrient	Content (%)
Corn	51.95	Crude protein (CP)	21.50
Soybean	39.50	Methionine (Met)	0.50
Rapeseed oil	4.10	Calcium (Ca)	1.00
dl-Metionine	0.20	All phosphorus (P)	0.70
Calcium hydrogen phosphate	1.85	Methionine + cysteine (Met + Cys)	0.84
Calcium carbonate	1.30	Lysine (Lys)	1.15
Sodium chloride	0.40	Threonine (Thr)	0.83
Trace element premix ^a^	0.50	Metabolizable energy (ME) (MJ/Kg)	29.90
Choline	0.17		
Multivitamins ^b^	0.03		
Total	100		

Trace element premix ^a^ (mg/kg): FeSO_4_·7H_2_O, 530; CuSO_4_·5H_2_O, 30; MnSO_4_·H_2_O, 400; ZnSO_4_·7H_2_O, 470; KI, 18; NaSeO_3_, 0.3; multivitamins ^b^: Vitamin A, 13,500 IU/kg; Vitamin D, 3000 IU/kg; Vitamin E, 24 IU/kg; Vitamin K_3_, 3 mg/kg; pantothenic acid, 15 mg/kg; folic acid, 1.05 mg/kg; nicotinamide, 30 mg/kg; biotin, 0.14 mg/kg.

### 4.2. Determination of the Proportion of T-Cell Subsets in the Intestine by FCM

#### 4.2.1. Isolation of the Intra-Epithelial Lymphocytes (IELs)

IELs were isolated as previously described by Montufar-Solis *et al.* [37]. Briefly, six broilers in each group were euthanized at 7, 14 and 21 days. The duodenal, jejunal and ileum samples were collected according to Yunus’ method [34]: Duodenum was defined as the segment encompassing duodenal loop and ileum was defined as the segment before the ileocecal junction equaling the length of the ceca. Jejunum was defined as the segment between the duodenum and ileum. The intestine was opened longitudinally, placed in a 100 mm tissue culture dish containing supplemented RPMI-1640 (Catalog No. SH4007-13, LOT: MXL0747; Hyclone, Logan, UT, USA). The sample tissue and media were transferred to a 50 mL plastic conical centrifuge tubes. After tissue pieces had settled, the medium was removed by suctioning and replaced with 15–20 mL of Ca^2+^ and Mg^2+^ free phosphate buffered saline (PBS), followed by 50 mL of Ca^2+^ and Mg^2+^ free PBS containing 2 mM DTT (CAS NO: 3483-12-3, Guangzhou Howei Chemical, Guangzhou, China) and 5 mM EDTA (CAS NO: 64-02-8; 13235-36-4, Taizhou Greenland Chemical, Zhejiang, China). The tissue suspension was transferred to a 100 mL beaker and stirred gently at 37 °C for 30 min. The tissue slurry was passed successively through two 10 cc syringe barrels filled to 5 mL with wetted nylon wool in order to remove undigested tissue pieces. The cell suspension was separated equally into two 50 mL tubes, and centrifuged for 10 min at 400× *g*. Cell pellets were mixed in 3 mL of 40% isotonic Percoll (Lot: 10036869, GE Healthcare Bio-Sciences AB, Uppsala, Sweden) (4 parts 100% Percoll and 6 parts 10× DPBS) and layered onto 4 mL of 70% isotonic Percoll (7 parts 100% Percoll and 3 parts 10× DPBS). The samples were centrifuged at 400× *g* for 30 min. Cells were collected from the two 40%/70% interface areas, combined and washed by centrifugation in supplemented RPMI-1640. The cell pellet was resuspended in 3 mL of 40% isotonic Percoll and overlayed onto 4 mL of isotonic 70% Percoll, and centrifuged at 400× *g* for 30 min. IELs were collected from the 40%/70% Percoll interface and washed by centrifugation in supplemented RPMI-1640. The IELs concentration was adjusted to 1.0 × 10^6^ cells/mL with phosphate buffered saline (PBS) and prepared for the methods of flow cytometry (FCM).

#### 4.2.2. Isolation of Lamina Propria Lymphocytes (LPLs)

According to the method described by Resendiz-Albor *et al.* [38], after EDTA treatment (as described in the isolation of IELs), the duodenal, jejunal and ileum segments were washed twice with 25 mL RPMI-1640 medium and then transferred to a 50 mL tube containing 25 mL of RPMI-1640 with 60 U/mL of type IV collagenase (Sigma Chemical, St. Louis, MO, USA), 1% Fetal calf serum (FCS) and 50 µL/mL gentamicin. The tubes were incubated horizontally at 37 °C for 30 min in a shaking-water bath. The contents of each tube were transferred to petri dishes and 200 µL FCS were added. The intestinal mucosa was compressed with a syringe plunger over a plastic mesh. Single cell suspensions containing lamina propria cells were filtered through organdy mesh and then centrifuged 10 min at 2500× *g*. Cell suspensions were collected and centrifuged in a discontinuous 40/70% Percoll gradient at 600× *g* for 30 min. Cells collected from the interface were washed and suspended in RPMI-1640 medium with 1% FCS, and then centrifuged at 200× *g* for 5 min. The cell density was diluted to 1.0 × 10^6^ cells/mL with PBS and prepared for the methods of FCM.

#### 4.2.3. Flow Cytometry (FCM) Method

The aforementioned cell suspension (1 mL) was transferred to a centrifuge tube and centrifuged at 200× *g* for 5 min. The supernatant was discarded, and the cells were stained with 10 µL mouse anti-chicken CD3-SPRD (CatNo: 8200-13, LOT: H8905-MD60Y; BD Pharmingen, Franklin Lakes, NJ, USA), CD4-PIT (CatNo: 8210-02, LOT: J056-VG75C; BD Pharmingen, Franklin Lakes, NJ, USA) and CD8a-RPE (CatNo: 6220-09, LOT: E083-V805Y; BD Pharmingen, Franklin Lakes, NJ, USA) for 15–20 min at RT. After washing with PBS, the cells were resuspended in 0.5 mL PBS, and the percentages of T-cell subsets were determined by a BD FACSCalibur flow cytometer (BD Co., Ltd., Franklin Lakes, NJ, USA).

### 4.3. Determination of Cytokines mRNA Expression Levels in the Intestinal Mucosa by qRT-PCR

According to the methods described by Chen *et al.* [9], the mRNA expression of the IL-2, IL-4, IL-6, IL-10, IL-17, INF-γ and TNF-α (LITAF) were determined. At 7, 14, and 21 days of the experiment, the duodenal, jejunal and ileum samples from six chickens in each group were stored in liquid nitrogen, respectively. After adding liquid nitrogen, the intestines were crushed with a pestle into powder and filled into EP tubes immediately, then stored at −70 °C for future usage. Total RNA was extracted from the powder by TriPure isolation reagent (Roche Diagnostics GmbH, Mannheim, Germany). The quality of RNA (A260/A280) was 1.6–2.0 by spectrophotometric analysis and equalized by dilution in RNAase-free water. The mRNA was then reverse transcribed into complementary DNA (cDNA) using Transcription First Strand cDNA Synthesis (Roche Diagnostics GmbH, Mannheim, Germany). The cDNA was used as a template for quantitative real-time PCR analysis (qRT-PCR).

**Table 2:** Primer sequences, corresponding accession numbers and sizes of the amplification products.

Gene	Primer	Sequences (5'–3')	Product Size (bp)	Accession Number
IL-2	F	CCCGTGGCTAACTAATCTGC	114	AF000631
	R	TTGAGCCCGTAGGTTACAGAA		
IL-4	F	TCTTCCTCAACATGCGTCAG	108	NM_1007079.1
	R	GGTCTGCTAGGAACTTCTCCAT		
IL-6	F	CAAGGTGACGGAGGAGGAC	254	AJ309540
	R	TGGCGAGGAGGGATTTCT		
IL-10	F	CGGGAGCTGAGGTGAA	272	AJ621614
	R	GTGAAGAAGCGGTGACAGC		
IL-17	F	CAGATGCTGGATGCCTAACC	123	AJ493595
	R	CCAGTGAGCGTTTGCTGATA		
INF-γ	F	AGCTGACGGTGGACCTATTATT	259	Y07922
	R	GGCTTTGCGCTGGATTC		
TNF-α (LITAF)	F	TGTGTATGTGCAGCAACCCGTAGT	229	AY765397
	R	GGCATTGCAATTTGGACAGAAGT		
β-Actin	F	TGCTGTGTTCCCATCTATCG	150	L08165
	R	TTGGTGACAATACCGTGTTCA		

For qRT-PCR reactions, 20-μL mixtures were made by using FastStart Essential DNA Green Master (Roche Diagnostics GmbH, Mannheim, Germany) containing 10 µL FastStart Universal SYBR Green Master (ROX), 0.6 µL forward, 0.6 µL reverse primer, 6.8 µL RNAase-free water and 2 µL cDNA. Reaction conditions were set to 10 min at 95 °C (first segment, one cycle), 10 s at 95 °C and 30 s at Tm of a specific primer pair (second segment, 44 cycles) followed by 10 s at 95 °C, and 72 °C for 10 s (dissociation curve segment) using a Thermal Cycler (Step One Plus, Applied BioSystems, Foster City, CA, USA). Gene expression was analyzed, and β-actin was used as an internal control gene [39,40]. Sequence of primers was obtained from GenBank of NCBI. Primers were designed with Primer 5 and synthesized by Sangon Biotech (Shanghai, China) (Table 2). The control broilers responses (mRNA amount) were as reference values for between groups comparison within the same control day in each week, respectively. The qRT-PCR data were analyzed with 2^−∆∆*C*t^ calculation method of Livak and Schmittgen [41].

### 4.4. Statistics

The significance of difference between two groups was analyzed by variance analysis, and results presented as means ± standard error. The analysis was performed using the independent sample *t* test of SPSS software for Mac v.20.0 (IBM Corp, Armonk, NY, USA) and a value of *p* < 0.05 was considered significant.

## 5. Conclusions

In conclusion, this study has demonstrated that dietary 0.6 mg/kg the AFB_1_ in broilers reduced the percentages of the CD3^+^, CD3^+^CD4^+^ and CD3^+^CD8^+^ T-cell subsets in IELs and LPLs as well as the expression levels of the IL-2, IL-4, IL-6, IL-10, IL-17, IFN-γ and TNF-α mRNA in the mucosa of the duodenum, jejunum and ileum, implying that the immune function of the intestinal mucosa might be affected. Reduced mRNA expression of cytokines may be closely associated with the decreased proportions of T cells subsets induced by AFB_1_.

## References

[1] Lewis L., Onsongo M., Njapau H., Schurz-Rogers H., Luber G., Kieszak S., Nyamongo J., Backer L., Dahiye A.M., Misore A. (2005). Aflatoxin contamination of commercial maize products during an outbreak of acute aflatoxicosis in eastern and central Kenya. Environ. Health Perspect..

[2] Golli-Bennour E.E., Kouidhi B., Bouslimi A., Abid Essefi S., Hassen W., Bacha H. (2010). Cytotoxicity and genotoxicity induced by aflatoxin B_1_, ochratoxin A, and their combination in cultured Vero cells. J. Biochem. Mol. Toxicol..

[3] Meissonnier G., Marin D., Galtier P., Bertin G., Taranu I., Oswald I., Mengheri E., Mengheri E., Roselli M., Britti M.S., Finamore A. (2006). Modulation of the immune response by a group of fungal food contaminant, the aflatoxins. Nutrition and Immunity.

[4] Rawal S., Kim J.E., Coulombe R. (2010). Aflatoxin B_1_ in poultry: Toxicology, metabolism and prevention. Res. Vet. Sci..

[5] Raisuddin S., Singh K., Zaidi S., Paul B., Ray P. (1993). Immunosuppressive effects of aflatoxin in growing rats. Mycopathologia.

[6] Dugyala R.R., Sharma R.P. (1996). The effect of aflatoxin B_1_ on cytokine mRNA and corresponding protein levels in peritoneal macrophages and splenic lymphocytes. Int. J. Immunopharmacol..

[7] Meissonnier G.M., Pinton P., Laffitte J., Cossalter A.M., Gong Y.Y., Wild C.P., Bertin G., Galtier P., Oswald I.P. (2008). Immunotoxicity of aflatoxin B_1_: Impairment of the cell-mediated response to vaccine antigen and modulation of cytokine expression. Toxicol. Appl. Pharmacol..

[8] Tomkova I., Sevcikova Z., Levkut M., Revajova V., Conkova E., Laciakova A., Lenhardt L. (2002). Effect of aflatoxin B_1_ on CD3 T cells and alkaline phosphatase in the intestine of mice. Mycopathologia.

[9] Chen K., Shu G., Peng X., Fang J., Cui H., Chen J., Wang F., Chen Z., Zuo Z., Deng J. (2013). Protective role of sodium selenite on histopathological lesions, decreased T-cell subsets and increased apoptosis of thymus in broilers intoxicated with aflatoxin B_1_. Food Chem. Toxicol..

[10] Chen K., Yuan S., Chen J., Peng X., Wang F., Cui H., Fang J. (2013). Effects of sodium selenite on the decreased percentage of T cell subsets, contents of serum IL-2 and IFN-γ induced by aflatoxin B_1_ in broilers. Res. Vet. Sci..

[11] Chen K., Peng X., Fang J., Cui H., Zuo Z., Deng J., Chen Z., Geng Y., Lai W., Tang L. (2014). Effects of dietary selenium on histopathological changes and T cells of spleen in broilers exposed to aflatoxin B_1_. Int. J. Environ. Res. Public Health.

[12] Cukrova V., Kurita N., Akao M. (1992). An early effect of aflatoxin B_1_ administered in vivo on the growth of bone marrow CFU-GM and the production of some cytokines in rats. Mycopathologia.

[13] Qian G., Tang L., Guo X., Wang F., Massey M.E., Su J., Guo T.L., Williams J.H., Phillips T.D., Wang J.S. (2014). Aflatoxin B_1_ modulates the expression of phenotypic markers and cytokines by splenic lymphocytes of male F344 rats. J. Appl. Toxicol..

[14] Jakab G.J., Hmieleski R.R., Zarba A., Hemenway D.R., Groopman J.D. (1994). Respiratory aflatoxicosis: Suppression of pulmonary and systemic host defenses in rats and mice. Toxicol. Appl. Pharmacol..

[15] Li Y., Ma Q.G., Zhao L.H., Wei H., Duan G.X., Zhang J.Y., Ji C. (2014). Effects of lipoic acid on immune function, the antioxidant defense system, and inflammation-related genes expression of broiler chickens fed aflatoxin contaminated diets. Int. J. Mol. Sci..

[16] Bondy G.S., Pestka J.J. (2000). Immunomodulation by fungal toxins. J. Toxicol. Environ. Health B.

[17] Lillehoj H.S., Chung K.S. (1992). Postnatal development of T-lymphocyte subpopulations in the intestinal intraepithelium and lamina propria in chickens. Vet. Immunol. Immunopathol..

[18] Wittig B., Zeitz M. (2003). The gut as an organ of immunology. Int. J. Colorectal. Dis..

[19] Blaschitz C., Raffatellu M. (2010). Th17 cytokines and the gut mucosal barrier. J. Clin. Immunol..

[20] Kolars J.C., Benedict P., Schmiedlin Ren P., Watkins P.B. (1994). Aflatoxin B_1_-adduct formation in rat and human small bowel enterocytes. Gastroenterology.

[21] Huang A., Shibata E., Nishimura H., Igarashi Y., Isobe N., Yoshimura Y. (2013). Effects of probiotics on the localization of T cell subsets in the intestine of broiler chicks. J. Poult. Sci..

[22] Yarru L., Settivari R., Gowda N., Antoniou E., Ledoux D., Rottinghaus G. (2009). Effects of turmeric (*Curcuma longa*) on the expression of hepatic genes associated with biotransformation, antioxidant, and immune systems in broiler chicks fed aflatoxin. Poult. Sci..

[23] Marin D., Taranu I., Bunaciu R., Pascale F., Tudor D., Avram N., Sarca M., Cureu I., Criste R., Suta V. (2002). Changes in performance, blood parameters, humoral and cellular immune responses in weanling piglets exposed to low doses of aflatoxin. J. Anim. Sci..

[24] Bruneau J.C., Stack E., O’Kennedy R., Loscher C.E. (2012). Aflatoxins B_1_, B_2_ and G_1_ modulate cytokine secretion and cell surface marker expression in J774A. 1 murine macrophages. Toxicol. In Vitro.

[25] Girish C., Smith T. (2008). Impact of feed-borne mycotoxins on avian cell-mediated and humoral immune responses. World Mycotoxin J..

[26] Schroder K., Hertzog P.J., Ravasi T., Hume D.A. (2004). Interferon-γ: An overview of signals, mechanisms and functions. J. Leukoc. Biol..

[27] Bogdan C., Mattner J., Schleicher U. (2004). The role of type I interferons in non-viral infections. Immunol. Rev..

[28] Chung H.L., Hwang J.B., Park J.J., Kim S.G. (2002). Expression of transforming growth factor β1, transforming growth factor type I and II receptors, and TNF-α in the mucosa of the small intestine in infants with food protein–induced enterocolitis syndrome. J. Allergy Clin. Immunol..

[29] Blanchard C., Durual S., Estienne M., Bouzakri K., Heim M.H., Blin N., Cuber J.C. (2004). IL-4 and IL-13 up-regulate intestinal trefoil factor expression: Requirement for STAT6 and de novo protein synthesis. J. Immunol..

[30] Akira S., Hirano T., Taga T., Kishimoto T. (1990). Biology of multifunctional cytokines: IL 6 and related molecules (IL1 and TNF). FASEB J..

[31] Rothwell L., Young J.R., Zoorob R., Whittaker C.A., Hesketh P., Archer A., Smith A.L., Kaiser P. (2004). Cloning and characterization of chicken IL-10 and its role in the immune response to Eimeria maxima. J. Immunol..

[32] Abreu-Martin M.T., Targan S.R. (1996). Regulation of immune responses of the intestinal mucosa. Crit. Rev. Immunol..

[33] Yunus A.W., Razzazi-Fazeli E., Bohm J. (2011). Aflatoxin B_1_ in affecting broiler’s performance, immunity, and gastrointestinal tract: A review of history and contemporary issues. Toxins (Basel).

[34] Yunus A., Ghareeb K., Abd-El-Fattah A., Twaruzek M., Böhm J. (2011). Gross intestinal adaptations in relation to broiler performance during chronic aflatoxin exposure. Poult. Sci..

[35] National Research Council (NRC) (1994). Nutrient Requirements of Poultry.

[36] Kaoud H.A. (2013). Innovative methods for the amelioration of aflatoxin (AFB_1_) effect in broiler chicks. Sci. J. Appl. Res..

[37] Montufar-Solis D., Klein J.R. (2006). An improved method for isolating intraepithelial lymphocytes (IELs) from the murine small intestine with consistently high purity. J. Immunol. Methods.

[38] Resendiz-Albor A.A., Esquivel R., Lopez Revilla R., Verdin L., Moreno-Fierros L. (2005). Striking phenotypic and functional differences in lamina propria lymphocytes from the large and small intestine of mice. Life Sci..

[39] Shini S., Kaiser P. (2009). Effects of stress, mimicked by administration of corticosterone in drinking water, on the expression of chicken cytokine and chemokine genes in lymphocytes. Stress.

[40] Hong Y.H., Lillehoj H.S., Lillehoj E.P., Lee S.H. (2006). Changes in immune-related gene expression and intestinal lymphocyte subpopulations following *Eimeria maxima* infection of chickens. Vet. Immunol. Immunopathol..

[41] Livak K.J., Schmittgen T.D. (2001). Analysis of relative gene expression data using real-time quantitative PCR and the 2^−ΔΔ*C*t^ method. Methods.

